# Extracellular Vesicles Derived From *Ex Vivo* Expanded Regulatory T Cells Modulate *In Vitro* and *In Vivo* Inflammation

**DOI:** 10.3389/fimmu.2022.875825

**Published:** 2022-06-22

**Authors:** Aaron D. Thome, Jason R. Thonhoff, Weihua Zhao, Alireza Faridar, Jinghong Wang, David R. Beers, Stanley H. Appel

**Affiliations:** Department of Neurology, Houston Methodist Neurological Institute, Houston Methodist Research Institute, Houston Methodist Hospital, Houston, TX, United States

**Keywords:** exosome (vesicle), inflammation, immune modulation, neuroinflammation, peripheral inflammation, inflammatory disease, neurodegenerative disease

## Abstract

Extracellular vehicles (EVs) are efficient biomarkers of disease and participate in disease pathogenesis; however, their use as clinical therapies to modify disease outcomes remains to be determined. Cell-based immune therapies, including regulatory T cells (Tregs), are currently being clinically evaluated for their usefulness in suppressing pro-inflammatory processes. The present study demonstrates that *ex vivo* expanded Tregs generate a large pool of EVs that express Treg-associated markers and suppress pro-inflammatory responses *in vitro* and *in vivo*. Intravenous injection of Treg EVs into an LPS-induced mouse model of inflammation reduced peripheral pro-inflammatory transcripts and increased anti-inflammatory transcripts in myeloid cells as well as Tregs. Intranasal administration of enriched Treg EVs in this model also reduced pro-inflammatory transcripts and the associated neuroinflammatory responses. In a mouse model of amyotrophic lateral sclerosis, intranasal administration of enriched Treg EVs slowed disease progression, increased survival, and modulated inflammation within the diseased spinal cord. These findings support the therapeutic potential of expanded Treg EVs to suppress pro-inflammatory responses in human disease.

## Introduction

Extracellular vesicles (EVs) were originally proposed as a mechanism for cells to dispose of damaged organelles, proteins, and nucleic acids (1, 2). However, additional evidence suggests that EVs can participate in active intercellular communication *via* transmission of signaling cargo (3, 4). These packages are now studied for their ability to modify cellular processes in health and disease (5–8). EVs are membrane-encapsulated particles ranging from approximately 20 nm to 1000nm and are released by cells into the extracellular space. EVs use signaling proteins, enzymes, coding and non-coding RNA (mRNA, microRNA, long noncoding RNA, etc.), DNA, surface proteins and receptors, lipids, and glycoproteins for intercellular signaling (9–11). These EV signaling packages can be further delineated by their size as well as their biogenesis. Specifically, exosomes (~20-200nm) are packaged and generated through the endosomal pathway *via* fusion of multivesicular bodies with the plasma membrane. Micro/nanovesicle populations (~50-1000nm) result from the outward budding and fission of the plasma membrane. Larger apoptotic bodies (~1-5 microns in size) are formed during the late stages of apoptosis as part of cell shrinkage and associated programmed cell death (12–16). Uptake and delivery of these EV packages and their cargo are accomplished through multiple recipient cell mechanisms such as membrane fusion, endocytosis, ligand-receptor interactions, antigen presentation, phagocytosis, macropinocytosis, lipid-raft, and more (17–19). EV distribution and communication among cells in an organism is both local and systemic.

EV functions can be deleterious or beneficial. Deleterious actions can reflect disease states and/or drive disease pathology such as disease-associated misfolded proteins, dysfunctional mitochondria, oxidative stressors, pro-inflammatory immune signaling components, and signaling promoting cellular injury and death (5, 20–26). Conversely, EVs derived from cells such as mesenchymal stem cells and anti-inflammatory immune cells produce EVs that promote cellular health with the potential to resolve inflammatory injury and cell stress (27–33). Thus, EVs derived from anti-inflammatory cells have potential therapeutic value in modifying disease-associated inflammatory states that promote disease and progression.

The immune system plays an important role in maintaining homeostasis and responding to disease pathogens. Dysfunction in one or more parts of the immune system can promote unwanted inflammation and autoimmune disease. There is now substantial evidence suggesting that the immune system plays an integral part in the pathogenesis and progression of neurodegenerative diseases such as Alzheimer’s (AD), Parkinson’s (PD), Multiple Sclerosis (MS) and amyotrophic lateral sclerosis (ALS) with accompanying dysfunction in regulatory T cells (Tregs) (34–41). Targeting immune dysfunction and the resulting inflammation has been clinically successful in autoimmune diseases such as multiple sclerosis and is presently being studied in neurodegenerative diseases (42–44). In ALS, Tregs have been shown to be dysfunctional; *ex vivo* expansion of these Tregs can correct their dysfunctional suppression and provide clinical benefit (35, 45, 46). In a phase I clinical trial, the autologous infusions of expanded Tregs into patients with ALS were found to be safe and well-tolerated, and slowed disease progression during early and later stages of disease (47). EVs derived from Treg cells have been proposed as a promising potential therapeutic approach for some time, but a better understanding of their immunomodulatory functions and mechanisms is still needed to advance them to the clinic (48, 49). In the present study, EVs were isolated from *ex vivo* expanded Tregs and assayed for their anti-inflammatory function both *in vitro* and *in vivo*. The generation and isolation of a large pool of EVs from Treg expansions that retain the parent cells’ suppressive functions may provide a novel therapeutic platform.

## Methods

### Expansion Protocols for Generating Treg EVs

The mixed Treg EVs were derived from media byproduct of ALS patient Treg expansions or control patient Treg expansions. Leukapheresis products were collected from ALS patients at the Blood Donor Center of Houston Methodist Hospital according to internal procedures or from Gulf Coast Regional Blood Center. For the ALS expanded process prior to their use in their clinical trial, Treg cells were isolated and cultured in the University of Texas Health Science Center at Houston (UTHealth) in the Judith R. Hoffberger Cellular Therapeutics Laboratory that is a FDA-registered, CAP- and FACT-accredited current Good Manufacturing Practice (cGMP) facility. For the control patient expanded Tregs, isolation and expansion protocols were done in the research setting while following the clinical trial expansion protocols and operations to produce a consistent, reproducible, and sterile process. For the cell isolations, patients’ CD4+CD25+ Tregs were isolated from their leukapheresis products through an initial CD8+ and CD19+ cell depletion step (CD8+ and CD19+ depletion reagent, Miltenyi Biotec) followed by a CD25+ cell enrichment step (CD25+ enrichment reagent, Miltenyi Biotec) using the automated CliniMACS Plus System (Miltenyi Biotec). Treg cells from the isolation process were then cultured in *ex vivo* Treg expansions. Tregs were expanded in TexMACS GMP medium containing 1% human AB serum, MACS GMP ExpAct Treg (CD3/CD28 beads, Bead:cell=4:1), IL-2 (500IU/ml) and Rapamycin (100 nM). Culture of the Tregs was performed in a TerumoBCT Quantum Bioreactor. IL-2 and Rapamycin were replenished every 2-3 days. Restimulation of Tregs was performed by adding MACS GMP ExpAct Treg (CD3/CD28 beads, Bead:cell=1:1) at day 15 only if needed to reach the dose of Tregs necessary for the clinical trial. After 15-25 days in culture, supernatants of Treg cultures were collected and stored at -20 C for EV isolation at a later date. Note that this Treg expansion process utilized a 1% serum supplement during the expansion process which results in a mixture of EVs derived from the Treg cell expansions and the media serum supplement. Control population of media EVs was generated by isolating EVs from the unused TexMACS media with the 1% serum supplement added. Media EVs are used as a control for the differential effects of the Treg EV mixed population being analyzed.

For the experiments using Treg EVs without the serum EVs (enriched Treg EVs), we replicated the smaller-scale expansion protocol used by the lab in previous studies but added the inclusion of exosome-depleted serum supplement instead of the normal serum supplement (36, 38). CD4+CD25+ Tregs were isolated from healthy control blood using the Human CD4+CD25+ Regulatory T Cell Isolation Kit (Miltenyi Biotec) according to the manufacturer’s instructions. Tregs were expanded in TexMACS GMP medium containing 5% exosome-depleted FBS (Gibco), MACS GMP ExpAct Treg (CD3/CD28 beads, Bead:cell=4:1), IL-2 (500IU/ml) and Rapamycin (100 nM). IL-2 and Rapamycin were replenished every 2-3 days. Restimulation of Tregs was performed by adding MACS GMP ExpAct Treg (CD3/CD28 beads, Bead:cell=1:1) at day 15. After 25 days in culture, supernatant from the Treg culture was collected and stored at -20 C for EV isolation at a later date.

### Extracellular Vesicle Isolations

Treg EVs were isolated using either the polyethylene glycol precipitation method (PEG) that was initially used for small-scale studies or *via* tangential flow filtration (TFF) that was later optimized for larger scale EV isolations (50). ExoQuick-TC reagent (System Biosciences, SBI) was used for the PEG isolations and protocol conducted according to manufacturer’s instructions. Briefly, media from Treg expansion cultures were centrifuged at 3000 x g for 15 minutes to remove cells and debris. PEG reagent was added to spun supernatant at 1:5 ratio of PEG: tissue culture media, mixed thoroughly, and refrigerated overnight at 4C. Mixture was then centrifuged at 1500 x g for 30 minutes, supernatant aspirated, spun again at 1500 x g for 10 minutes, and supernatant aspirated again. EV pellet was resuspended in sterile PBS and diluted for nanoparticle analysis using Nanosight NS300 nanoparticle analyzer for Treg EV size/concentration analysis. EVs were stored at -20C while limiting freeze/thaw cycles. For TFF isolations, Treg expansion media was collected and processed through a KrosFlo K2Ri TFF system (Repligen/Spectrum) *via* a two-step process for the isolation, concentration and diafiltration of Treg EVs. Step 1 utilized a Midi 20cm 0.65um mPES 0.75mm hollow fiber filter (Repligen) for the elimination of any cells, beads, and/or debris from the tissue culture media. Step 1 permeate was used as the input for further processing in Step 2. Step 2 utilized a Midi 20cm 500kD mPES 0.5mm hollow fiber filter (Repligen) for the concentration of Treg EVs in the retentate while soluble material smaller than the filter passed through to the permeate. Following concentration, a 10X diafiltration step took place with the same filter/setup in order to wash and buffer exchange the Treg EVs into sterile PBS solution. Final concentrated Treg EV product is found in the Step 2 retentate, and aliquots of final and intermediate products were obtained for nanoparticle analysis using the Nanosight NS300 nanoparticle analyzer for size distribution, particle concentration, and TFF EV yield.

### Nanosight EV Size/Concentration Readings

EV readings were obtained using Nanosight NS300 (Malvern Panalytical) particle analyzer. EV samples were optimally diluted to appropriate concentrations for readings and analyzed using continual measurement at a constant flow of 50 (arbitrary units) with 3 recordings/analyses of 60 seconds each with the following parameters: camera level (12–15), temperature (22C), and detection threshold (5). Concentration was recorded as particles/ml and size statistics were recorded as mean and mode.

### Treg EV Surface Characterization

Surface protein expression on the EV populations was assessed using the MACSPlex Exosome Kit (Miltenyi Biotec) according to manufacturer’s instructions and analyzed on MACSQuant Analyzer flow cytometer (Miltenyi Biotec). Briefly, EV populations were incubated overnight with a cocktail of various fluorescently labeled bead populations coated with specific antibodies targeting different surface epitopes. Exosome detection reagents are used to form sandwich complexes on the beads that are then analyzed based on their unique fluorescent characteristics. Distinct positive populations can then be measured with the MACSQuant flow cytometer.

### iPSC-derived M1 Myeloid Cultures

Induced pluripotent stem cell (iPSC) derived myeloid cells were used to obtain a consistent inflammatory response independent of any patient-specific confounding factors. Pro-inflammatory iPSC-derived myeloid cells were generated using protocols previously developed/described and recapitulated over time by our lab for multiple studies (36, 38, 39, 51). Briefly, a 4-step culturing process allows for the generation of CD14+ from control patient iPSC lines. CD14 myeloid cells are isolated using positive magnetic selection with Miltenyi Biotec CD14 beads, isolation columns, and MACS magnetic cell separator. For differentiation into M0 macrophages, CD14 cells are cultured in complete RPMI media (10% fetal bovine serum, 25mM HEPES, 1mM sodium pyruvate, 1xnonessential amino acids, 55uM 2-mercaptoethanol, 100 units/ml penicillin, and 100ug/ml streptomycin) supplemented with 50 ng/ml GMCSF (R&D systems) for 7 days to create M0 cells for M1 use. M0 cells are then primed with 0.1ng/ml Lipopolysaccharide (LPS) (Sigma) and 0.2 ng/ml IFN-γ (Invitrogen) in order to polarize myeloid cells to become pro-inflammatory (M1).

### Treg EV Suppression Assays With Myeloid Cells and Tresp Proliferation Assays

M0 (GMCSF) cells are detached using enzyme-free dissociation buffer, pelleted, and plated at a density of 50,000 cells/well in 96 well, flat bottom plates. M1 cells are primed with 0.1ng/ml LPS (Sigma) and 0.2 ng/ml IFN-γ (Invitrogen) for 1 hour to polarize to M1 cells. Treg EVs (1x10^8^ particles) are added into cultures following M1 polarization for overnight time point (~18 hrs) followed by collection of the confitioned media for M1-derived cytokine/protein analysis. Supernatants were collected from co-culture groups and IL-6 protein amounts were assessed using ELISA-based immunoassays (Invitrogen). Pro-inflammatory output is measured in IL-6 production by M1 cells and normalization of suppression in the figures is compared to max output of IL-6 from pro-inflammatory M1 cells without treatment. For Tresp proliferation assays, Tresp cells from an allogeneic control are isolated using Miltenyi Biotec magnetic bead/column reagents and protocols to negatively isolate CD4+CD25- T cells (Tresps) from control patient peripheral blood samples. Tresps are plated at 50,000 cells per well in 96 well, round-bottom plates and stimulated with CD3/28 beads (Miltenyi Biotec). Treg EVs are added to the cultures in escalating doses and remain in Tresp culture through co-culture experiment. Following 4 days in culture, Tresps are pulsed with tritium and proliferation is determined by examining tritium incorporation 18 hours after tritium pulsing. Results of the assay are reported as the percent inhibition of Tresp proliferation compared to Tresps that are activated but not treated with EVs.

### LPS-Induced Neuroinflammation Mouse Model and SOD1 Mouse Model of ALS

All animal experimental procedures were approved by the Houston Methodist Research Institute’s Institutional Animal Care and Use Committee in compliance with the National Institutes of Health guidelines. All mice were monitored for pain, distress, and adverse effects from EV treatments. For the acute LPS-induced neuroinflammation mouse model, C57Bl6 WT mice were injected intraperitoneally (IP) with 2mg/kg LPS (Sigma; O111:B4) followed by peripheral (IV) or intranasal administration of various Treg EV doses after 2 hours post-LPS injection. IV administration of Treg EVs were dosed with either 1x10^9^, 1x10^10^, or 1x10^11^ particles *via* tail vein injection and intranasal administration of enriched Treg EVs were given 1x10^9^ particles. Dosing paradigms were within effective dose ranges previously reported (52). Mice were then sacrificed at 14-16 hours post model initiation followed by removal of organs for RNA analysis, specifically spleens for further immune cell isolation/analysis and different regions of the brain. Immune cells were isolated from freshly isolated spleens by extracting single cell suspension using a 40um cell strainer followed by magnetic, bead-based isolation techniques for myeloid cells (CD11b+ isolation kit; Miltenyi Biotec) and T cell populations (CD4+CD25+ isolation kit; Miltenyi Biotec). Isolated immune cells were flash frozen for subsequent RNA isolation and transcript analysis using RT-PCR. For CNS tissue, mouse brains were freshly isolated and different regions of the brain were dissected on ice and flash frozen for RNA isolation and transcript analysis using RT-PCR.

For the ALS mouse model, we utilized transgenic mice harboring the SOD1-G93A mutation that previously was described as a motor neuron degeneration model for ALS (53). SOD1 mice began phenotype assessments starting at day 70 followed by intranasal injections of enriched Treg EVs (1x10^9^ particles) beginning at day 90 at continuous intervals of every two weeks until they reached their ethically defined endpoint. Mouse phenotype was assessed using a modified “BASH scoring system” whereby SOD1 mice gain a degenerative point from 0 (no symptoms) to 6 (ethical endpoint) as phenotype worsens with disease progression (54–56). The phenotypes assessed and points added are as follows but not necessarily in this order: +1 Tremulousness, +1 Gait abnormalities, +1 Hindlimb weakness/paresis, +1 Weight loss of more than 10% adult weight, +1 Spasticity to one or both hindlimbs, +1 Paralysis. When animals reach ethical endpoint, they are sacrificed and organs are harvested, RNA isolated, and RT-PCR done for transcript analysis. Specifically, diseased lumbar spinal cords were isolated from mice to examine pro-inflammatory transcripts using RT-PCR. Dosing information prior to sacrifice were as follows: Number of doses (PBS 4, 5, 5, 5; Treg EV 6, 4, 5, 5, 10, 10); Average doses (PBS 4.75; Treg EV 6.67); Median doses (PBS 5; Treg EV 5.5). Follow up days from first treatment to sacrifice: Average days (PBS 61.75; Treg EV 72.83); Median days (PBS 61.5; Treg EV 74.5); Minimum days (PBS 57; Treg EV 51); Maximum days (PBS 67; Treg EV 84).

### RNA Purification and RT-PCR Analysis

RNA was isolated from cells and tissues using Trizol reagent followed by Direct-zol RNA MiniPrep Plus Kit (Zymo Research) according to manufacturer’s recommendations. Quantitative RT-PCR was performed using one-step RT-PCR kit with SYBR Green (Bio-Rad) and an iQ5 Multicolor Real-Time PCR detection system (Bio-Rad). Primers for RT-PCR (IL-6, IL-1β, TNF, IL-10, Arg-1, IFN-γ, FOXP3, and CD206) were acquired from Bio-Rad and run according to the manufacturer’s protocols. The relative expression level of each mRNA was assessed using the ΔΔCt method and normalized to β-actin/controls.

### Protein Analysis Using ELISAs and Western Blot Analysis

Levels of CD73 (Abcam) and CTLA4 (Abcam) protein were measured by ELISA-based immunoassays according to manufacturer’s protocols. From the Treg EV and media EV preparations, a volume equivalent of 10ug of protein was added as input to each well of the ELISAs in triplicate for consistent protein normalization across samples. Treg cell protein from expanded Treg cells was also added in the same amount to directly compare to cell amounts. Following incubation protocols and washes, the immunoassay absorbances were read using a microplate plate reader at the assay’s specified wavelength.

Western blot analysis was used to identify Treg-related proteins in protein preparations from Treg EVs, medias EVs, and Treg cells. A volume equivalent of 30ug of protein was loaded as input for each well from the respective EV or cell sample onto a polyacrylamide gel (Biorad) and run at a voltage of 70 V for 30min and then changing to 120V for about an hour. Blots were transferred to nitrocellulose membrane, rinsed with Tris-buffered saline with 0.1% Tween (3X), and blocked with 6% powdered milk for 2 hours at room temperature. Subsequent steps required blots be incubated with 1:1000 dilution of either anti-CTLA4 (Abcam) or ICOS (Abcam) primary antibodies overnight at 4C followed by 1:2000 dilution of goat anti-rabbit secondary antibody at room temperature for 2 hours. Blots were then developed and exposed at various times to reveal optimal protein banding. Afterward, membranes were exposed to Ponceau S stain (ThermoFisher) for visual total protein normalization of the induvial sample lanes. Same protocols were used for negative control markers for EV isolation but with 1:500 dilution anti-Calnexin (ThermoFisher) or 1:1000 dilution of anti-GM130 (Abcam) followed by 1:2000 dilution goat anti-mouse secondary or goat anti-rabbit secondary, respectively.

### Treg Cell Flow Cytometry

Flow cytometry was used to assess Treg cells following isolation from leukapheresis product and following expansion protocols. Antibodies (anti-human) against the following targets were used in the staining and analysis: CD3 Alexa Fluor 700 (Invitrogen), CD4 V500 (BD Biosciences), CD8 eFluor 450 (Invitrogen), CD25 PerCP-Cy 5.5 (BD Biosciences), FOXP3 Alexa Fluor 488 (Invitrogen). Appropriate isotype controls were set for gating schemes and to establish background parameters. Live/Dead fixable blue dead cell UV stain kit was used to assess viability of the cells. For intracellular FOXP3 staining, cells were fixed and permeabilized using the FOXP3/Transcription Factor Staining Buffer Set (BD Biosciences) prior to staining. Cells were subsequently analyzed using a BD LSRII flow cytometer with BD FACSDIVA software.

## Results

### Treg EV Characterization

The isolation and *ex vivo* expansion of patient Treg cells generate an enriched CD4+CD25+ population of highly suppressive Treg cells (**Table 1**). Specifically, isolation protocols using the CliniMACS system initially depleted CD8+ and CD19+ cells followed by a CD25+ enrichment step that produced a CD4+CD25+ population that is greater than or equal to 70% (% of total CD4 population). *Ex vivo* expansion of these CD4+CD25+ cells results in a highly pure population of Treg cells typically reflected by a CD4+CD25+ population greater than or equal to 95% (% of total CD4+ population). Additionally, potential contaminating cell populations such as CD8+ and CD19+ cell populations remained below 0.07% when analyzed following a depletion step and below 1.5% following an enrichment step of the isolation (**Supplementary Table 1**). In addition to the high purity, we find that *ex vivo* expanded Treg cells are highly functional in their ability to suppress T cell proliferation with greater than 88% suppression of proliferation across patients at a 1:1 ratio (Treg: Tresp) (**Table 1**). These cells also express increased amounts of both CD25 and FOXP3 protein noted *via* flow cytometry analysis of MFI levels from CD4+CD25+ cells. The isolation and expansion protocols provide an enriched CD4+CD25+ suppressive Treg cell population from which EVs can be isolated.

**Table 1:** Treg cell characterization following isolation and expansion.

Treg Cell Characterization	CliniMACS cell isolation			Post Treg Expansion		
ALS Treg Expansion Cells	Starting cell population	Post CD8+ CD19+ cells depletion	Post CD25+ cells enrichment	Cells following Treg expansion and harvest		
ALS Treg Expansion 1	38.8	33.92	72.85	99.94	65.6
ALS Treg Expansion 2	37.4	49.95	80.31	98.92	63.1
ALS Treg Expansion 3	27.16	32.16	88.42	99.15	70.8
	**% CD4+CD25+ Treg (% of CD4+ population by flow cytometry)**			**%CD4+CD25+FOXP3+**		
**Treg Cell Characterization**	**Treg Suppression of Tresp Proliferation**			**Treg Cell protein via flow cytometry**		
**ALS Treg Expansion Cells**	**Treg Suppression at Baseline (%)**	**Treg Suppression after Expansion (%)**	**Treg CD25 MFI Baseline**	**Treg CD25 MFI after Expansion**	**Treg FOXP3 MFI Baseline**	**Treg FOXP3 MFI after Expansion**
ALS Treg Expansion 1	not available	88.1	466	9031	604	1448
ALS Treg Expansion 2	not available	86.1	532	9171	891	1017
ALS Treg Expansion 3	not available	94.5	502	27507	830	1793
**Treg Cell Characterization**	**CliniMACS cell Isolation**			**Post Treg Expansion**		
**Control Treg Expansion Cells**	**Starting cell population**	**Post CD8+ CD19+ cells depletion**	**Post CD25+ cells enrichment**	**Cells following Treg expansion and harvest**		
Control Treg Expansion 1	not available	not available	61.87	99.48	37.04
Control Treg Expansion 2	not available	not available	83.04	99.23	47.05
	**%CD4+CD25+ Tregs (% of CD4+ population by flow cytometry)**			**%CD4+CD25+FOXP3+**		
**Treg Cell Characterization**	**Treg Suppression of Tresp Proliferation**			**Treg Cell protein via flow cytometry**		
**Control Treg Expansion**	**Treg Suppression at Baseline (%)**	**Treg Suppression after Expansion (%)**	**Treg CD25 MFI Baseline**	**Treg CD25 MFI after Expansion**	**Treg FOXP3 MFI Baseline**	**Treg FOXP3 MFI after Expansion**
Control Treg Expansion 1	7.2	92.4	503	20633	442	3093
Control Treg Expansion 2	4.8	94	684	25850	482	1785

Treg EVs were derived from leukapheresis products from ALS and control patients. These products underwent an isolation procedure consisting of a depletion step of CD8+ and CD19+ cells following by an enrichment step for CD4+CD25+ cells. These cells were then used as input for the expansion cultures to activate and expand the Treg cell population. Data shows flow analysis of cell populations before isolation, after depletion step, after enrichment step, and following expansion protocols. Characterization is included for the suppressive activity of the Treg cells prior to expansion (baseline) and following expansion along with flow analysis of Treg protein MFI increases of both CD25 and FOXP3.

**Figure 1:**
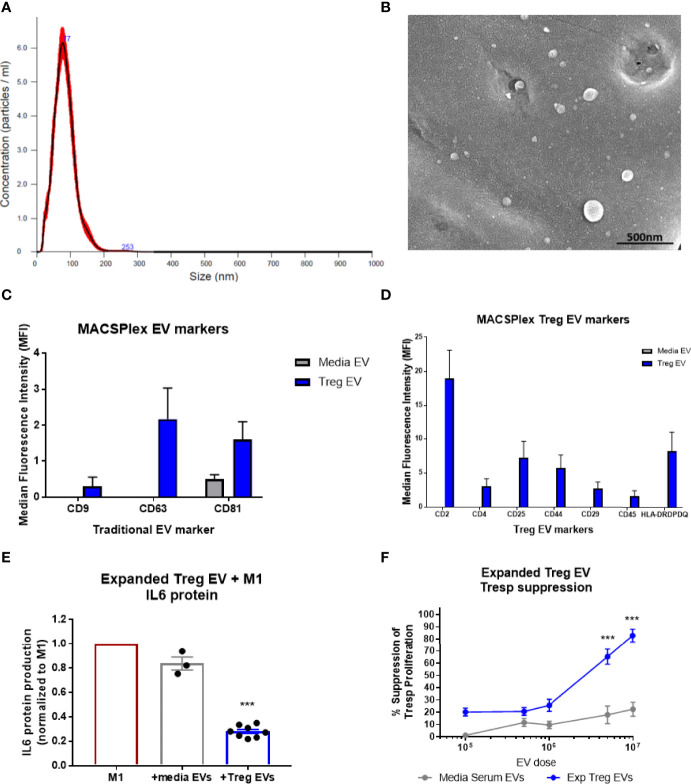
Characterization of Treg EVs isolated from Treg cells and *in vitro* suppressive function. **(A)** Nanoparticle analysis of Treg EVs demonstrates single peak distribution within a 20-200nm range consistent with functional EV subsets such as exosomes and microvesicles. **(B)** SEM imaging confirms EV particles within the range of the nanoparticle analysis. **(C, D)** Miltenyi MACSPlex exosome analysis shows Treg EVs are positive for common exosome markers of CD9, CD63, and CD81 while media EVs from added serum are only positive for CD81. Additional MACSPlex exosome assay results differentially define the Treg EV population as being positive for CD2, CD4, CD25, CD44, CD29, CD45 and HLA-DRDPDQ compared to the media EVs from supplemented serum components that express none of these markers. **(E)** *In vitro* treatment of pro-inflammatory myeloid cells (M1) with Treg EVs from ALS patient expanded Tregs reduces the IL-6 protein following overnight co-culture treatment. Suppression of IL-6 production with EV treatments normalized to max IL-6 output of M1 cells with activation and no treatment. **(F)** Escalating doses of Treg EVs from expanded Tregs differentially suppress Tresp proliferation compared to media EVs. Numbers shown as averages ± SEM with analysis *via* one-way ANOVA with Tukey’s *post hoc* testing (***p < 0.001). (**A, B**: representative data/images. **C, D**: Treg EVs n = 6 and media EVs n = 3. **E, F**: Treg EVs n = 7 and media only EVs n = 3).

During the expansion process of the CD4+CD25+ cells following isolation, EV-enriched media is generated and collected as a byproduct. EVs from the byproduct were isolated, initially *via* polyethylene glycol (PEG) precipitation and later by tangential flow filtration (TFF) for scale, and analyzed using Nanosight nanoparticle analysis. The nanoparticle analysis of the EVs isolated during the expansion of diverse Treg populations demonstrated a consistent size distribution between 50nm and 150nm (Mean= 94.5nm, Mode= 76.8nm, D10 = 56.6nm, D50(Median)= 86.4nm, D90 = 146.9nm) (**Figure 1A**). Scanning electron microscopy confirmed Treg vesicle size and shape (**Figure 1B**). These Treg EVs were found to express the combination of confirmed vesicle surface markers of CD9, CD63, and CD81; media EVs only expressed the CD9 vesicle marker (**Figure 1C**). Additionally, these Treg EVs were positive for surface markers of CD2, CD4, CD25, CD44, CD29, CD45, and HLA-DRDPDP; media EVs did not express any of these markers (**Figure 1D**). Treg EVs derived from expansion of both ALS patients and controls show the same vesicle and Treg-conserved markers when run using the MACSPlex exosome assay (**Supplementary Figures 1C, D**). Characterization of the Treg EVs demonstrate a size distribution consistent with an EV definition along with unique markers that are differentially expressed compared to the media EVs. Along with characterization of the EVs, purity of the EV isolations were assessed using suggested markers of contamination that are not of plasma membrane or endosomal origin, specifically GM130 and calnexin (**Supplementary Figure 3**) (57, 58). Treg EVs from ALS patients and controls along with media EVs did not express either GM130 or calnexin while the Treg cells did suggesting purity of the EV populations.

### Treg EV Suppressive Functions *In Vitro*

*In vitro* co-culture of Treg EVs with pro-inflammatory myeloid cells (M1) and T cells were performed to test suppressive capacity. The Treg EVs suppressed pro-inflammatory iPSC-derived M1 cell IL-6 protein production at 1x10^8^ particles per 50,000 activated M1 cells; media EVs used as the differential control demonstrated very little suppressive function (**Figure 1E**). Treg EVs in increased doses were added to responder T cells (Tresp) resulting in a dose-dependent inhibition of Tresp proliferation. At a dose of 5x10^6^ particles, suppression was 65% and suppression increased to 82% at a dose of 1x10^7^ (**Figure 1F**). At higher doses than 1x10^7^ EVs, we found increased media EV suppression that looks to be a culturing artifact; potentially derived from large quantities of EVs disrupting Tresp and CD3/CD28 bead interactions in the smaller wells of the 96-well plate that the assay is optimized for. Differential isolation techniques using either PEG or TFF isolations have no consequence on Treg EV suppressive function (**Supplementary Figures 1A, B**). Additionally, a direct suppressive functional comparison between Treg EVs mesenchymal stem cell EVs (MSC EVs) demonstrated that Treg EVs are more suppressive in co-culture with both pro-inflammatory myeloid cells and Tresp proliferation (**Supplementary Figures 1E, F**). Overall, Treg EVs derived from *ex vivo* expanded Treg cells demonstrated a unique and Treg-conserved signature along with suppressive function *in vitro* that was comparable to expanded Treg cells.

### *In Vivo* Effects of Treg EVs in an Acute LPS-Induced Mouse Model of Inflammation

**Figure 2:**
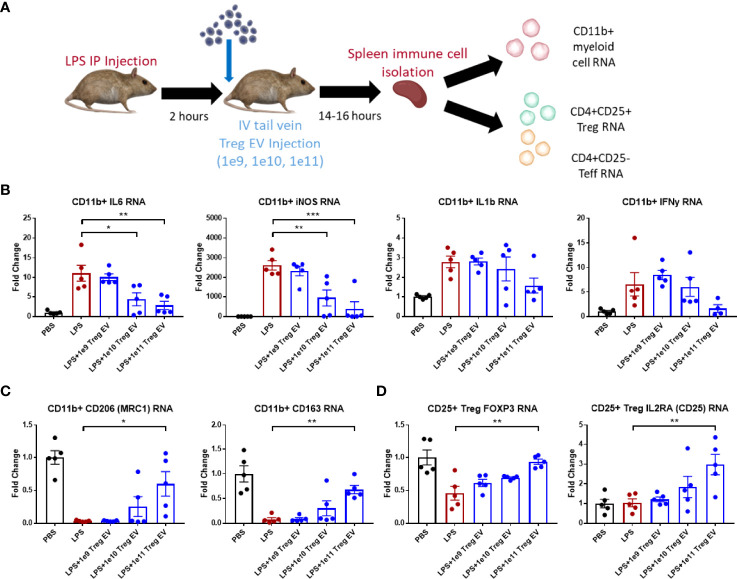
Treg EVs suppress pro-inflammatory myeloid cells and T cell proliferation. **(A)** Model of LPS-induced model of acute inflammation whereby 2mg/kg of LPS is given IP to WT mice and subsequently treated with single tail vein (IV) injections of different doses of Treg EVs. Following overnight treatment, animals were sacrificed, and peripheral immune cells were freshly isolated from mice spleens for subsequent inflammatory transcript analysis. **(B)** Isolated CD11b+ myeloid cells from the spleen demonstrated a dose-dependent reduction in pro-inflammatory transcripts of IL-6, iNOS, IL-1β, and IFNγ following IV treatment of Treg EVs in the LPS-induced inflammatory model of disease. **(C)** Additionally, CD11b+ myeloid cells increased their production of anti-inflammatory associated transcripts of CD206 (MRC1) and CD163 following treatment with Treg EVs. **(D)** Fresh spleen isolated CD4+CD25+ Tregs demonstrated increased Treg health and function trasncripts of FOXP3 and IL2RA (CD25) following Treg EV treatment. Data shown in **Figure 2** represents averages ± SEM and statistical analysis done with one-way ANOVA analysis with Tukey’s *post hoc* test (**Figure 2** groups: PBS n = 5, LPS n = 5, LPS+Treg EV 1x10^9^ n = 5, LPS+Treg EV 1x10^10^ n = 5, LPS+Treg EV 1x10^11^ n = 5). P-values are *p < 0.05, **p < 0.01, and ***p < 0.001.

Treg EVs were administered *via* tail vein injection (IV) at different doses in mice following 2 mg/kg IP LPS injection to induce inflammation (**Figure 2A**). Following overnight time point after 14-16 hours, mice were sacrificed, and spleens dissected to isolate immune cells including CD11b+ myeloid cells, CD4+ CD25+ Treg cells, and CD4+ CD25- effector T cells. Transcript analysis of spleen-derived CD11b+ myeloid cells showed an induction of pro-inflammatory transcripts such as IL-6, iNOS, IL-1β, and IFN-γ following LPS-induced inflammation induction in Treg EV untreated mice (**Figure 2B**). Increasing peripheral doses of Treg EVs demonstrated a corresponding reduction in spleen-derived myeloid pro-inflammatory IL-6 transcripts at doses of 1x10^10^ (61%) and 1x10^11^ (75%) while also reducing iNOS transcripts in these same cells at doses of 1x10^10^ (64%) and 1x10^11^ (85%). A decreasing trend in IL-1β and IFN-y transcripts was observed with the best results at the higher doses administered. Anti-inflammatory transcripts were assayed from these myeloid cells following LPS activation and subsequent Treg EV peripheral treatment. Additionally, Treg EVs at a dose of 1x10^11^ particles induced the production of anti-inflammatory transcripts such as MRC1 (mannose receptor/CD206) and CD163 in these pro-inflammatory myeloid cells compared with LPS injected only animals (**Figure 2C**).

T cell populations were also isolated from the spleens of these animals to assess the immune-modulating effects of the Treg EVs on CD4+CD25+ Treg populations and CD4+CD25- effector T cell (Teff) populations. The Treg population demonstrated a decrease in the FOXP3 expression following LPS-induced inflammation. Treatment with Treg EVs increased FOXP3 transcript levels in a dose-dependent fashion with an increase in expression at the 1x10^11^ dose (**Figure 2D**). Additionally, an increase in IL2RA transcripts (CD25) was observed at the highest Treg EV doses. The CD4+CD25- Teff cells did not exhibit activation in multiple inflammatory transcripts investigated at this time point including TNF-α and IFN-y. With respect to the Treg EV treatments, there were no observable inflammatory or anti-inflammatory immune transcript alterations or cell proliferation in the Teff cell population following Treg EV treatment.

### IV Administration of Treg EVs Produces Modest Neuroinflammatory Changes in the Brain

**Figure 3:**
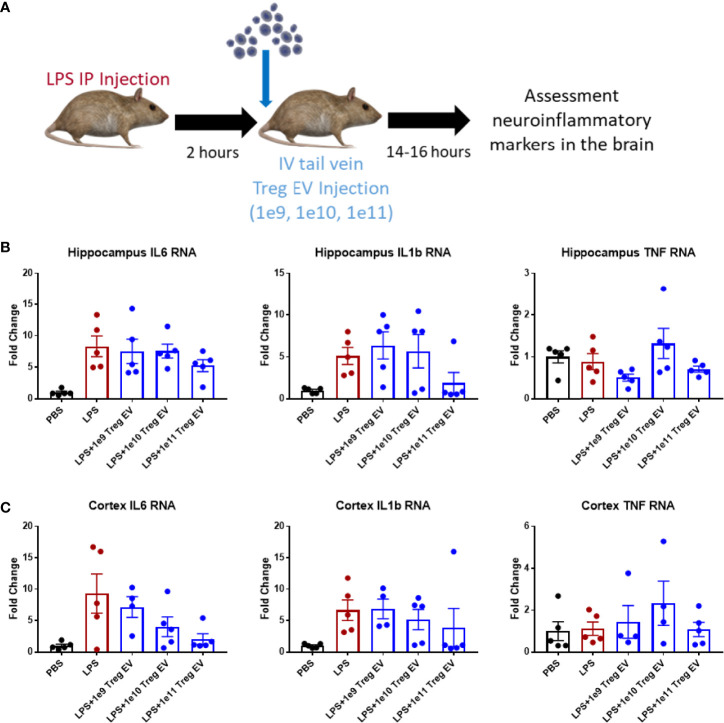
Treg EVs suppress pro-inflammatory transcripts in LPS-induced model of neuroinflammation. **(A)** Model of LPS-induced model of acute inflammation whereby 2mg/kg of LPS is given IP to WT mice to induce and assess neuroinflammation followed by subsequent single tail vein (IV) injections of different doses of Treg EVs. Following overnight treatment, animals were sacrificed, and CNS brain tissue was freshly isolated for inflammatory transcript analysis. **(B)** Hippocampal inflammatory RNA analysis following IV Treg EV treatment demonstrated a modest decrease in pro-inflammatory IL-6 and IL-1β transcripts at high doses. **(C)** Analysis of cortex tissue showed a similar modest suppression of pro-inflammatory IL-6 and IL-1β following Treg EV treatment. We did not observe significant TNF RNA changes in the hippocampus or cortex. Data shown in **Figure 3** represents averages ± SEM and with one-way ANOVA analysis with Tukey’s *post hoc* testing (**Figure 3**: PBS n = 5, LPS n = 5, LPS+Treg EV 1x10^9^ n = 4-5, LPS+Treg EV 1x10^10^ n = 4-5, LPS+Treg EV 1x10^11^ n = 5).

Since IV administration of Treg EVs modulates peripheral immune cell signatures in the LPS-induced model of inflammation, the extent of neuroinflammatory immune modulation in inflamed mice following IV administration of Treg EVs was evaluated. In the same treated animals, brain regions were dissected, and transcript analysis was performed to assess the potential immunomodulation of peripheral Treg EVs in reducing neuroinflammation (**Figure 3A**). In the hippocampus of the affected mice, IV delivered Treg EVs produced only a modest reduction in pro-inflammatory transcripts of IL-6 while IL-1β had a mixed result; both cytokines were reduced at the highest dose of 1x10^11^ particles (**Figure 3B**). In the cortex, there was a trend for a dose-dependent suppression of IL-6 and IL-1β (**Figure 3C**). TNF-α transcripts in both regions were not elevated in LPS-induced animals.

### Intranasally Administered Treg EVs Suppress Neuroinflammation in LPS-Induced Inflammation

**Figure 4:**
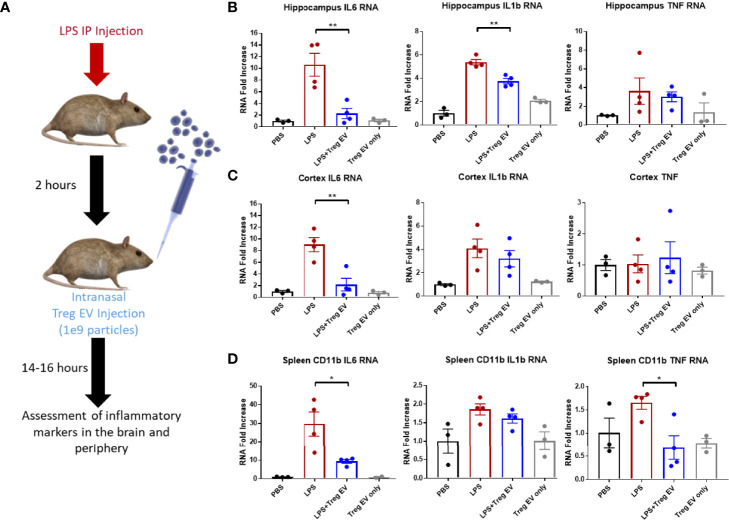
Enriched Treg EVs given intranasally suppress pro-inflammatory transcripts in LPS-induced model of neuroinflammation. **(A)** Neuroinflammation was initiated using 2mg/kg peripheral LPS IP injection followed by intranasal administration of 1x10^9^ enriched Treg EVs or PBS control. Inflammatory transcript analysis from CNS and peripheral tissue samples was done following overnight treatment. **(B)** Hippocampal RNA analysis showed a decrease in pro-inflammatory IL-6 and IL-1β RNA in enriched Treg EV treated mice when compared with LPS plus intranasal PBS treated animals. **(C)** Evaluation of cortex tissues showed a reduction in IL-6 transcripts following enriched Treg EV treatment. We did not observe significant TNF RNA changes in the hippocampus or cortex regions while IL-1β was not reduced in the cortex. **(D)** In the periphery, CD11b+ spleen-derived myeloid cells demonstrated decreased IL-6 and TNF-α transcripts following treatment with intranasal enriched Treg EVs. Notably, Treg EV only treatments without LPS induction did not show increased proinflammatory transcripts in CNS or peripheral tissues with any of the provided parameters during the experiments. **Figure 4** data shown as averages ± SEM and with one-way ANOVA analysis with Tukey’s *post hoc* test (**Figure 4**: PBS n = 3, LPS+PBS IN n = 4, LPS+Treg EV IN n = 4, Treg EV IN only n = 3). P-values are *p < 0.05 and **p < 0.01.

Tregs were expanded utilizing an exosome-depleted serum to obtain a population of enriched Treg EVs. Mice were injected with LPS peripherally to induce peripheral and central nervous system inflammation followed by intranasal administration of 1x10^9^ enriched Treg EVs (**Figure 4A**). Enriched Treg EVs suppressed hippocampal IL-6 and IL-1β transcripts generated by the LPS injections; there was no change in TNF-α transcripts following treatment (**Figure 4B**). In examining the cortex, there was a treatment-specific reduction in IL-6 transcripts while IL-1β and TNF-α transcripts remained elevated (**Figure 4C**). Modulation of peripheral inflammation was assayed by analyzing inflammatory transcript changes in splenic-derived CD11b+ myeloid cells. Interestingly, there was a robust decrease in myeloid IL-6 and TNF-α transcripts following intranasal enriched Treg EV treatment (**Figure 4D**). These results suggest an enhanced immune-modulating response of neuroinflammation following treatment with enriched Treg EVs given intranasally.

### Enriched Treg EVs Suppress Inflammation and Extend Survival in a Mouse Model of ALS

**Figure 5:**
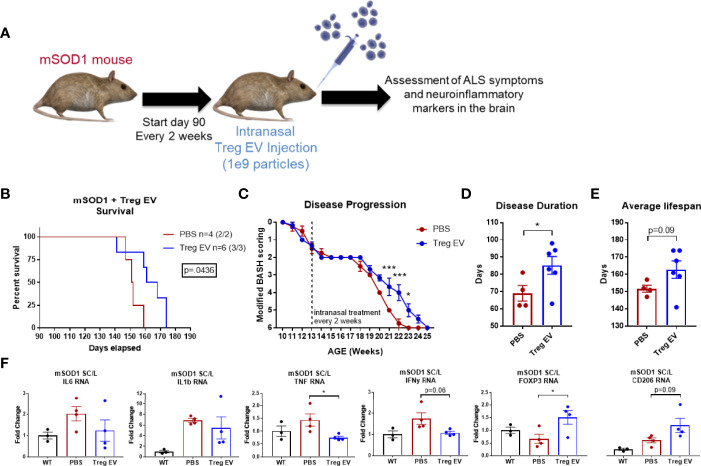
Enriched Treg EVs given intranasally increases survival, slows disease progression, and modifies neuroinflammation in mSOD1 treated mice. **(A)** mSOD1 mice were treated intranasally with 1x10^9^ particles of enriched Treg EVs or PBS every 2 weeks starting at day 90 until ethical endpoint is reached. **(B)** Treatment with enriched Treg EVs increased overall survival when compared to PBS control treated mice. **(C)** Disease progression was assessed using a modified “BASH” scoring system developed to analyze deficits in mice with motor neuron disease. Enriched Treg EV treatment delayed disease progression in later, rapid progressing stages of disease. **(D)** Disease duration from first symptom to ethical endpoint was increased in enriched Treg EV treated animals compared with PBS controls. **(E)** The average lifespan of mSOD1 mice treated with enriched Treg EVs is increasing compared to PBS controls. **(F)** Once mice reach ethical endpoints, diseased lumbar spinal cord tissues were harvested for analysis of inflammatory transcripts. Enriched Treg EV treatments reduced inflammatory transcripts in the lumbar spinal cord of mSOD1 mice; statistically reducing TNF transcripts and trending with IL-6, IL-1β, and IFN-γ. Additionally, enriched Treg EV treatments increased anti-inflammatory Treg-associated FOXP3 transcripts and anti-inflammatory myeloid cell associated transcripts of CD206 (MRC1). Survival comparison analyzed and reported using Log-rank (Mantel-Cox test) and disease progression using two-way ANOVA with Sidak’s multiple comparisons test. Disease duration and lifespan analyzed using Welch’s t test (**B–D**: PBS n = 4 (2/2 M/F), Treg EV n = 6 (3/3 M/F).Transcript analysis of isolated lumbar spinal cord tissues analyzed using one- way ANOVA with Tukey’s multiple comparisons test (**F**: (PBS n = 4 (2/2 M/F), Treg EV n = 2 (2/2 M/F). P-values are *p < 0.05 and ***p < 0.001.

An ALS mouse model for motor neuron degeneration and the accompanying inflammatory responses was used to examine the effects of chronic intranasal administration of enriched Treg EVs on reducing neuroinflammation and associated disease. Intranasal treatments of 1x10^9^ particles of enriched Treg EVs were initiated at day 90 when the animals were already showing signs of motor neuron disease (**Figure 5A**). Enriched Treg EV treatment significantly increased the survival in the treated group compared to PBS intranasally administered controls (**Figure 5B**). Additionally, we observed that the enriched Treg EV treatments slowed disease progression in the later, more rapid progressing stages of disease (54–56) (**Figure 5C**). When examining the disease duration from first symptom to end stage disease, enriched Treg EVs increased disease duration (symptom onset to sacrifice) to 85 days compared with disease duration of 69 days in the PBS treated mice (**Figure 5D**). Average lifespan of the treated animals was increased in the enriched Treg EV treated animals compared to PBS controls at 162.8 days vs 151.7 days, respectively (p=0.09) (**Figure 5E**). Additionally, follow-up time from first treatment to sacrifice increased in the Treg EV treated animals compared to controls (average days: PBS 61.75, Treg EV 72.83; median days: PBS 61.5, Treg EV 74.5; minimum days: PBS 57, Treg EV 51; maximum days: PBS 67, Treg EV 84). With respect to overall intranasal doses administered, mice treated with PBS ended with 4 to 5 intranasal doses with an average of 4.75 doses while the Treg EV treated group ultimately received 4 to 10 intranasal doses with an average dose of 6.67 in their group. Following animal end stage disease and subsequent sacrifice, we extracted RNA from the lumbar portions of their diseased spinal cord to evaluate treatment-associated inflammatory changes *via* transcript analysis (**Figure 5F**). Examination of pro-inflammatory transcripts found decreases in TNF-α and IFN-y transcripts in lumbar spinal cords of enriched Treg EV treated animals while IL-6 and IL-1β transcripts trended in the same decreasing direction (**Figure 5F**). Additionally, levels of FOXP3 transcripts were increased with Treg EV treatment; myeloid-specific CD206 transcripts were also increased in Treg EV treated ALS mice compared with PBS treated mice. Together, these data show that intranasal Treg EVs can increase survival, ameliorate motor neuron disease progression, decrease pro-inflammatory mechanisms in the spinal cord, and increase beneficial anti-inflammatory signatures indicative of increased Treg and anti-inflammatory myeloid cell contributions.

## Discussion

The development and utilization of immune cell-based therapeutics is an effective way to modulate inflammatory responses in animals and patients (59). The current study reinforces this concept by demonstrating that Treg EV particles can suppress peripheral and central nervous system (CNS) inflammation. First, these results suggest that Treg EVs isolated from media produced by *ex vivo* Treg expansions provide EV particles that are consistent in size and are positive for common exosome and Treg-associated markers. These particles were shown to robustly suppress pro-inflammatory myeloid cells and T cell proliferation *in vitro*. Second, IV administration of these Treg EVs modulated inflammation in an LPS-induced inflammatory mouse model by suppressing pro-inflammatory myeloid cells, promoting increased peripheral anti-inflammatory transcripts, and increasing Treg-associated markers. Third, anti-inflammatory suppressive functions were documented following intranasal administration of expanded Treg EVs in the LPS-induced inflammatory model suggesting an enhanced immune-modulating response of neuroinflammation following intranasal treatment with enriched Treg EVs. Lastly, the enriched Treg EV particles slowed disease progression, increased survival, and modulated disease-associated inflammation in the spinal cord in a mouse model of ALS. The cumulative results of this study suggest an exciting therapeutic potential for Treg EV treatment of neurodegeneration and other inflammation-related diseases.

The source Treg cells for expansions are derived from a leukapheresis product that undergoes negative and positive selection *via* a CliniMACS isolation system and reagents that first depletes CD8+ and CD19+ cells followed by an enrichment step for the CD25+ cell populations. This process provides an input material for the Treg expansion that contains highly viable cells that are depleted of B cells (CD19) and cytotoxic T cells (CD8) while enriched with CD25+ cells (**Supplementary Table 1**). The Treg expansion takes these enriched CD25+ cells and combines a cocktail treatment paradigm of IL-2, rapamycin, and CD3/CD28 activation beads to generate a large number of highly pure and immunosuppressive CD4+CD25+ Treg cells (**Table 1**). The Treg cells increase their CD25 and FOXP3 protein expression following expansion which are surrogate markers of Treg health and suppressive function. Most importantly, the Treg cells have robust suppressive function when examined in co-culture in T cell proliferation assays demonstrating a greater than 85% suppression of T cell proliferation at a 1:1 ratio of Tresp cells to Treg cells. This isolation and expansion process provides a characterized Treg cell population with robust suppressive capacity from which we isolate and test Treg EVs.

EVs are a combination of different vesicles that are batched into different groups according to their size, function, and biogenesis with smaller-medium vesicles such as exosomes and microvesicles providing essential intercellular communication through a variety of mechanisms while larger vesicles are associated with cell death products such as apoptotic bodies (13, 16). Isolation and sourcing of EVs can have perceived variable effects on the function and characterization parameters of the EV product. To account for these potential confounding variables, we directly compared the different isolation techniques used in our study. Initial isolation techniques for the Treg EVs utilized polyethylene glycol precipitation (PEG) but this technique proved difficult to scale up from the hundreds of milliliters to liters of media byproduct volume being generated. Development of a tangential flow filtration system (TFF) allowed for this scaled isolation, concentration, and diafiltration of Treg EVs (50). In assessing whether Treg EV suppressive function is different with different isolation techniques, we found no significant change in suppressive function on iPSC-derived pro-inflammatory myeloid cells or on T cell proliferation (**Supplementary Figures 1A, B**). Regarding Treg cell sourcing for expansions and subsequent Treg EVs from those expansions, our lab has performed multiple studies showing consistent characterization and immune-modulating function between expanded Treg cells from patients with neurodegenerative disease (ALS, AD, PD) and from age-matched controls (34, 36, 38, 46, 47). Direct comparisons of Treg EV characterization from the ALS patient-derived Treg EVs and the control-derived Treg EVs show the same common exosome markers of CD9, CD63, and CD81 (**Supplementary Figure 1C**) while also having the conserved Treg-associated markers of CD2, CD4, CD25, CD44, CD29, and HLA-DRDPDQ (**Supplementary Figure 1D**) as determined by MACSPlex exosome analysis. Changes in expression magnitude in these markers could be a result of optimization of the Treg expansion process or due to assay being more qualitative than quantitative in its reporting. Overall, the Treg expansion process described in this study yields both a consistent Treg cell and Treg EV product, regardless of the Treg cell sourcing from patients or EV isolation technique.

Treg EVs demonstrated *in vitro* suppressive function in their ability to suppress pro-inflammatory cells such as iPSC-derived pro-inflammatory myeloid cells and T cell proliferation. Previous studies have shown the ability of EVs derived from Tregs to suppress CD4+ and CD8+ T cell proliferation (60–62). One study rules out CTLA4 as the causative mechanism for this suppression and proposes CD73, both expressed on their Treg EVs, as the causative mechanism as blocking CTLA4 did not alter Treg EV suppression while their CD73-expressing Treg EVs could hydrolyze exogenous 5’-AMP to immunosuppressive adenosine at an equal rate to that of Tregs cells. Another study demonstrated that their Treg EVs contain enriched and unique microRNA networks compared to EVs derived from other T cells and that these microRNAs, specifically miR-Let-7d, contributes to the suppression of T cell proliferation. Inhibition of sphingolipids also showed an alteration in the immunosuppressive effects provided by the Treg EVs. These studies suggest that a combination or network of multiple mechanisms are packaged and transmitted by Treg EVs in a context dependent manner to different types of cells. A different study examined the effects Treg EVs on modifying dendritic cell function and found that their Treg EVs contain microRNAs that are transferred to these antigen presenting cells to cause a decrease in pro-inflammatory IL-6 protein and concurrent increase in anti-inflammatory IL-10 following LPS stimulation and Treg EV treatment (63). In line with these results, our experiments demonstrate the ability for Treg EVs to suppress iPSC-derived, pro-inflammatory myeloid cells. It should be noted that dosing paradigms for Treg EVs across these *in vitro* assays are within the ranges utilized in our studies. The mechanism(s) of action for the suppressive function of Treg EVs on both lymphoid and myeloid immune cell subsets is warranted and currently under investigation.

While this study does not specifically identify mechanism of action for the Treg EVs, characterization of proteins on or within the Treg EVs could point to the potential mechanisms at play. The MACSPlex exosome assay, which provided a number of Treg cell-conserved surface markers in its panel, demonstrated that the Treg EVs express Treg markers on their surface. The quintessential surface markers for Treg cells involve expression of CD4 and CD25 that were prominently expressed on the Treg EVs. Additionally, CD44 has previously documented to be expressed on Treg cells and positively correlate with FOXP3 expression and Treg suppressive function (64, 65). CD29 is a cell surface receptor found on Tregs that is hypothesized to be associated with expression of CD73 and CTLA4 and their well-known immune-modulating functionality in Treg cells. Expression of HLA constituents on Treg cells resulted in far more suppressive function of the Treg cell compared than their negative counterparts (66–68). Other additional proteins involved with Treg and their suppressive mechanisms were found in our study on the resulting Treg EVs through various other assays including CTLA4, CD73, and ICOS (**Supplementary Figure 2**) (69–71). Specifically, CTLA4 was confirmed *via* western blot and ELISA analysis in Treg EVs from ALS and control isolated and expanded Tregs while media EVs showed no expression using the same protein input. CD73 were detected *via* ELISA in the Treg EVs without a signal from the media EV populations. Continued examination of the mechanisms of Treg EV immune modulation of both myeloid cells and T cells is warranted.

The *in vivo* results using Treg EVs suggest an anti-inflammatory effect in a model of inflammation and a motor neuron degenerative disease model of ALS. EVs have been administered previously into preclinical animal models of disease with efficiency and effectiveness, particularly in influencing the peripheral immune system (72–77). IV administration of the Treg EVs demonstrated a robust ability to dampen pro-inflammatory transcripts in peripheral CD11b+ myeloid cells. The increase in anti-inflammatory transcripts in these same cells suggest possible Treg EV-induced repolarization of the peripheral myeloid cells. Additionally, isolated Tregs increased known markers of FOXP3 and IL2RA (CD25) suggesting amplification of the Treg cells and their function. Although immune-modulating benefits were seen in the periphery *via* IV treatment of Treg EVs, we were unable to document a significant decrease in inflammatory transcripts within the brain *via* this route of administration. Previous studies show anti-inflammatory and restorative benefits of EVs injected *via* tail vein injections in a number of neuroinflammatory and neurodegenerative diseases (78–82).

In a strategy to directly target the CNS, we administered enriched Treg EV particles intranasally and observed CNS neuroinflammatory changes through reductions in pro-inflammatory transcripts in multiple areas of the brain. Intranasal administration of EVs provides a direct path to the CNS for neurological disease and has been documented in several animal models of disease (83–88). In fact, tracking of EVs *via* gold-labeled nanoparticle tracking demonstrated that intranasal administered EVs tracked to specific areas of the brain and neuroinflammation (89, 90). Although neuroinflammatory benefits were seen, mechanisms of EV entry, localization, and function requires further investigation. With respect to the mSOD1 preclinical mouse model of ALS, we found that intranasal administration of enriched Treg EVs after symptom onset could slow disease progression and increase survival. Of interest, enriched Treg EVs have immunomodulatory effects in the diseased spinal cord of the mSOD1 mice and this effect is promising as a potential treatment for ALS and other neurodegenerative diseases. Notably, no significant difference was detected between male and female mice with these mSOD1 model outcomes. Treg cell therapies in ALS patients have demonstrated safety and tolerability while also showing potential signs of slowing disease progression, although larger studies need to be completed (47). Treg EVs present as a potential immune-modulating therapy that could harness the robust activity of a cell-based therapy but with far less risk. In this regard, Treg EVs could be used as either a stand-alone therapy or in conjunction with the Treg cells themselves.

In both IV and intranasal treatments using the EVs, we did not observe any indications of immunoreactivity or alloreactivity from the treatments in the mice. One of the benefits of utilizing EVs is that there is increased perceived therapeutic safety due to reduced immunogenicity and alloreactive risk and experimental findings suggesting as such (91–95). The *in vivo* experiments utilized human Treg EV injections into two different preclinical mouse models of disease without any observable immune reactivity or animal distress. However, a number of variables can influence potential toxicity including EV dose, cell source from which they are derived, frequency of administration, administration route, and more. We utilized a single administration of EVs in an acute model of inflammatory disease along with a repeated intranasal administration of an enriched Treg EV product for the chronic dosing in the mSOD1 model. Consideration of Treg EV therapeutic targets and administration approaches should be considered when dealing with a disease of the CNS, the periphery, or an interconnected combination of the two.

Treg cell-based therapies are a burgeoning class of potent, immune-modulating therapies being developed by many groups for the treatment of a variety of diseases from autoimmune and inflammatory disease to CNS-related neurodegenerative diseases. Although immune suppression is highly potent in the Treg cell-based products, limitations exist in the ability of Tregs to withstand an active, pro-inflammatory milieu and resist undergoing apoptosis or conversion to Th17 cells. Treg EVs have the ability not only to suppress pro-inflammatory immune cells *in vitro* and *in vivo*, but also to stabilize Tregs viability and function at least *in vitro* (data not shown). Clinical studies are presently being planned to determine whether Treg EVs could be utilized as a standalone therapy, or possibly used in combination with Treg cell-based therapies as a pre-treatment to reduce the pro-inflammatory milieu and/or as a frequent booster between Treg-therapy treatments to suppress the milieu and boost Treg health and function. Choosing the best parameters for EV treatment necessarily should consider disease pathogenesis, the route of administration, dosing concentration and frequency and more.

Much of the current therapeutic focus on EV implementation to the clinic revolves around using mesenchymal stem cell (MSC) derived EVs for disease treatment because of their proposed anti-inflammatory function seen in preclinical studies (32, 33, 96, 97). MSC EVs, in the research setting, demonstrate only modest suppressive function while MSC cells utilized clinically have historically shown limited efficacy suggesting that MSC EVs would have limited effects as an immune-modulating treatment (84, 98–103). In a direct comparison of patient derived MSC EVs and enriched Treg EVs in our *in vitro* suppression assays, we found that Treg EVs are far more potent than MSC EVs in suppressing pro-inflammatory myeloid cells and T cell proliferation (**Supplementary Figure 1E, F**). Consequently, EVs derived from immunosuppressive immune cells, such as Tregs, may demonstrate a more promising approach for combatting inflammation-associated diseases in translational studies and clinical implementation.

Overall, the results presented in this study demonstrate that EVs derived from immune cells, particularly Treg EVs, appear to maintain Treg cell characteristics and suppressive function. Technologies advancing Treg EV production, characterization, and isolation promote a potential and imminent clinical utility of Treg EVs to be used as an autologous therapeutic in a multitude of inflammation-associated diseases such as ALS and other neurodegenerative diseases.

## Data Availability Statement

The original contributions presented in the study are included in the article/**Supplementary Material**. Further inquiries can be directed to the corresponding author.

## Ethics Statement

The animal study was reviewed and approved by Houston Methodist Research Institute’s Institutional Animal Care and Use Committee.

## Author Contributions

AT and SA conceived and designed the research. AT and JW performed the experiments. AT, JT, WZ, AF, DB, and SA took part in the analysis and interpretation of the data. AT, DB, and SA wrote the manuscript with input from all authors. All authors have read and approve the final version of the manuscript.

## Funding

We are grateful to the Ann Kimball and John W. Johnson Center for Cellular Therapeutics and Coya Therapeutics, Inc. for support of this study. The funder was not involved in the study design, collection, analysis, interpretation of data, the writing of this article or the decision to submit it for publication.

## Conflict of Interest

AT reports consulting work with respect to Coya Therapeutics. SA is the chair of the scientific advisory board for Coya Therapeutics.

The remaining authors declare that the research was conducted in the absence of any commercial or financial relationships that could be construed as a potential conflict of interest.

## Publisher’s Note

All claims expressed in this article are solely those of the authors and do not necessarily represent those of their affiliated organizations, or those of the publisher, the editors and the reviewers. Any product that may be evaluated in this article, or claim that may be made by its manufacturer, is not guaranteed or endorsed by the publisher.

## Supplementary Material

Supplementary Figure 1 Additional characterization of Treg EVs. **(A, B)** EV isolation technique using either PEG isolations or TFF isolations does not affect the suppressive capabilities of Treg EVs in the M1 suppression assay or the suppression of T cell proliferation assay using the same samples but different isolation techniques (n=3 for each group). **(C)** Common exosome markers were positive in both the Treg EVs isolated from ALS patients and control patients (n = 3 for ALS Expanded Treg EVs and n = 6 for Control Expanded Treg EVs). **(D)** Treg EV associated markers are positive in both Expanded Treg EVs from ALS patients and Control patients (n = 3 for ALS Expanded Treg EVs and n = 6 for Control Expanded Treg EVs). These are positive EV surface markers that were differentially expressed compared to EVs derived from expansion media containing serum supplement. **(E)** Enriched Treg EVs are more suppressive of pro-inflammatory IL-6 protein produced by iPSC-derived M1 cells compared to MSC EVs. **(F)** Robust T cell proliferation inhibition by enriched Treg EVs compared to MSC EVs. Control EVs are derived from serum-containing supplement for expansion media. N = 3 per group and statistics run using one-way ANOVA with Sidak’s multiple comparisons testing ***p < 0.001.

Supplementary Figure 2 Characterization of Treg cell conserved proteins in Treg EVs. **(A)** Treg EVs derived from ALS and control expansions expressed CTLA4 protein compared to media EVs and Treg cells as visualized by western blot. **(B)** ICOS protein is visualized *via* western blot in Treg EVs compared to media EVs and Treg cells. Total protein normalization using Ponceau S staining below the blots was used to demonstrate equal protein input of 30ug total protein per sample per lane. **(C, D)** ELISA analysis of CD73 protein and CTLA4 protein in Treg EVs compared to media EVs. Expanded Treg cell protein used for comparison. Protein amounts are read as pg/mL concentration from 10ug protein input into each well.

Supplementary Figure 3 Purity EV preparations from expansions and isolations. Protein analysis of EV contaminating proteins in the isolated EV preps from the Treg expansions from control patients, ALS patients, and media only without culture. No signal of Golgi marker, GM130, or ER membrane marker, calnexin, in the Treg or media EV preps. Both signals are found in protein from expanded Treg cells. Total protein normalization using Ponceau S staining below the blots for equal protein input of 30ug total protein per sample per lane.

Supplementary Table 1 Depletion characteristics of Treg cells following isolation from leukapheresis products. Details of flow cytometry analysis immune cell populations following CD8+ and CD19+ depletion and CD25+ enrichment. Data shows a viable cell population from leukapheresis product with mixed cell population. Following depletion and enrichment steps during isolation, minimal amounts of CD8+ and CD19+ cells remain in the cell population.
