# Supplementary figures and images for: Extracellular Vesicles Derived From Ex Vivo Expanded Regulatory T Cells Modulate In Vitro and In Vivo Inflammation
Source: Front Immunol. 2022 Jun 22;13:875825. doi: 10.3389/fimmu.2022.875825 (PMC9258040; doi:10.3389/fimmu.2022.875825)

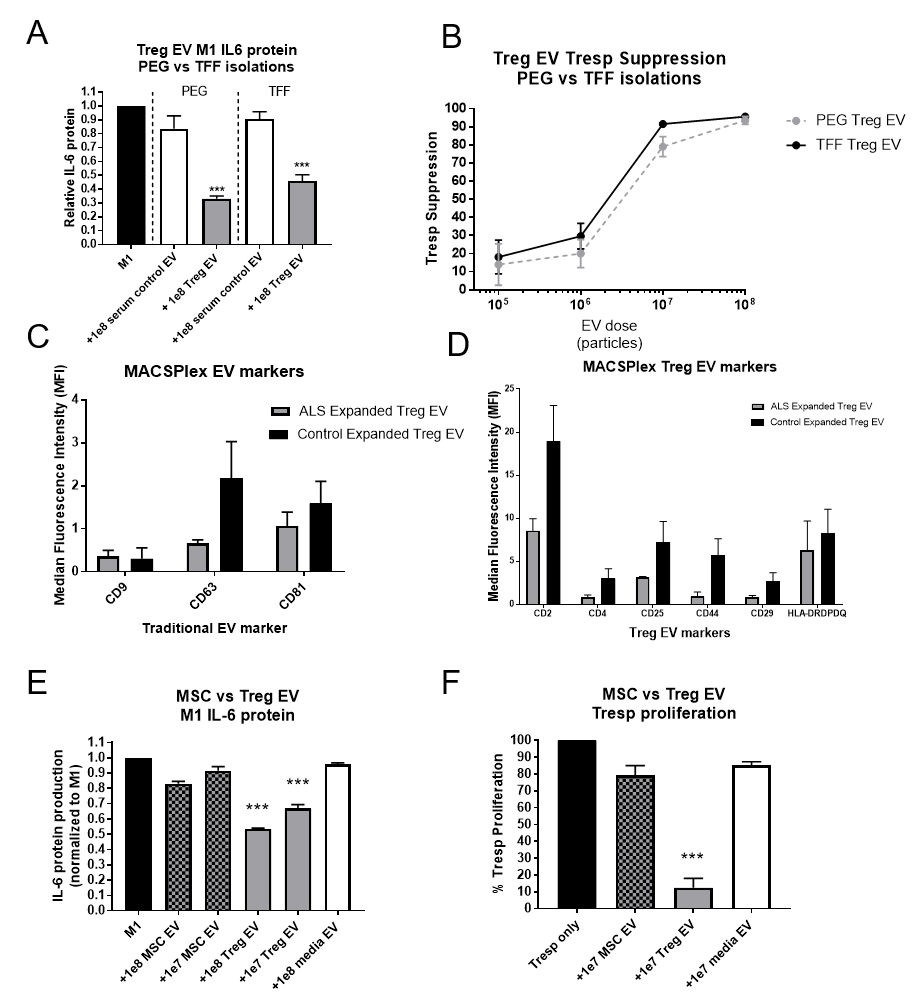

Supplement: Supplementary Figure 1 — Additional characterization of Treg EVs. (A, B) EV isolation technique using either PEG isolations or TFF isolations does not affect the suppressive capabilities of Treg EVs in the M1 suppression assay or the suppression of T cell proliferation assay using the same samples but different isolation techniques (n=3 for each group). (C) Common exosome markers were positive in both the Treg EVs isolated from ALS patients and control patients (n = 3 for ALS Expanded Treg EVs and n = 6 for Control Expanded Treg EVs). (D) Treg EV associated markers are positive in both Expanded Treg EVs from ALS patients and Control patients (n = 3 for ALS Expanded Treg EVs and n = 6 for Control Expanded Treg EVs). These are positive EV surface markers that were differentially expressed compared to EVs derived from expansion media containing serum supplement. (E) Enriched Treg EVs are more suppressive of pro-inflammatory IL-6 protein produced by iPSC-derived M1 cells compared to MSC EVs. (F) Robust T cell proliferation inhibition by enriched Treg EVs compared to MSC EVs. Control EVs are derived from serum-containing supplement for expansion media. N = 3 per group and statistics run using one-way ANOVA with Sidak’s multiple comparisons testing ***p < 0.001. [file Image_1.tif]

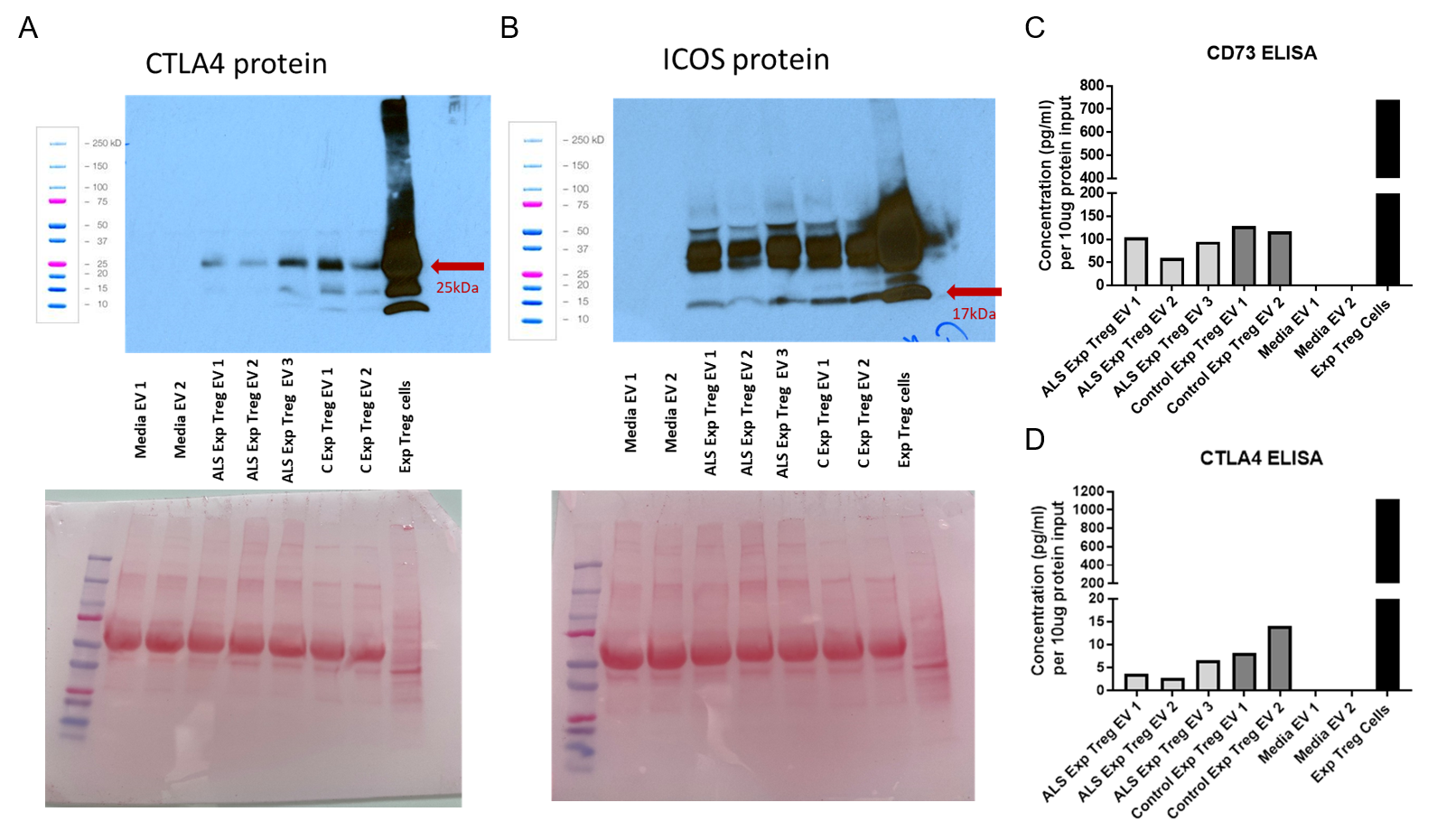

Supplement: Supplementary Figure 2 — Characterization of Treg cell conserved proteins in Treg EVs. (A) Treg EVs derived from ALS and control expansions expressed CTLA4 protein compared to media EVs and Treg cells as visualized by western blot. (B) ICOS protein is visualized via western blot in Treg EVs compared to media EVs and Treg cells. Total protein normalization using Ponceau S staining below the blots was used to demonstrate equal protein input of 30ug total protein per sample per lane. (C, D) ELISA analysis of CD73 protein and CTLA4 protein in Treg EVs compared to media EVs. Expanded Treg cell protein used for comparison. Protein amounts are read as pg/mL concentration from 10ug protein input into each well. [file Image_2.tif]

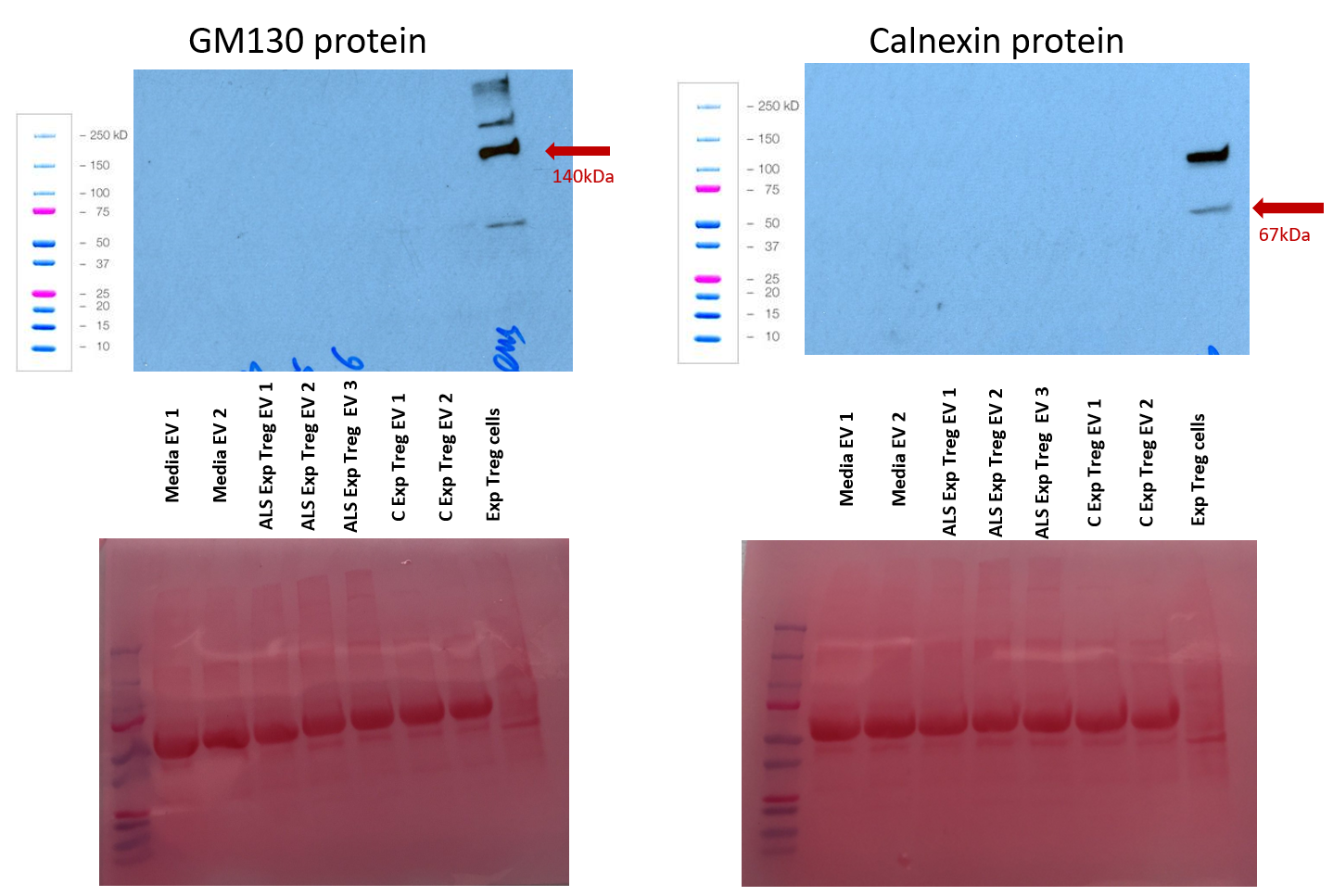

Supplement: Supplementary Figure 3 — Purity EV preparations from expansions and isolations. Protein analysis of EV contaminating proteins in the isolated EV preps from the Treg expansions from control patients, ALS patients, and media only without culture. No signal of Golgi marker, GM130, or ER membrane marker, calnexin, in the Treg or media EV preps. Both signals are found in protein from expanded Treg cells. Total protein normalization using Ponceau S staining below the blots for equal protein input of 30ug total protein per sample per lane. [file Image_3.tif]
